# Key Role of Equilibrium HONO Concentration over Soil in Quantifying Soil–Atmosphere HONO Fluxes

**DOI:** 10.1021/acs.est.1c06716

**Published:** 2022-02-01

**Authors:** Fengxia Bao, Yafang Cheng, Uwe Kuhn, Guo Li, Wenjie Wang, Alexandra Maria Kratz, Jens Weber, Bettina Weber, Ulrich Pöschl, Hang Su

**Affiliations:** † Multiphase Chemistry Department, Max Planck Institute for Chemistry, Mainz 55128, Germany; ‡ Institute of Biology, University of Graz, Graz 8010, Austria; § Department of Precision Machinery and Precision Instrumentation, University of Science and Technology of China, Hefei 230026, China; ∥ Minerva Research Group, Max Planck Institute for Chemistry, Mainz 55128, Germany

**Keywords:** HONO, equilibrium HONO concentration ([HONO]*), soil, nitrogen cycling, flux

## Abstract

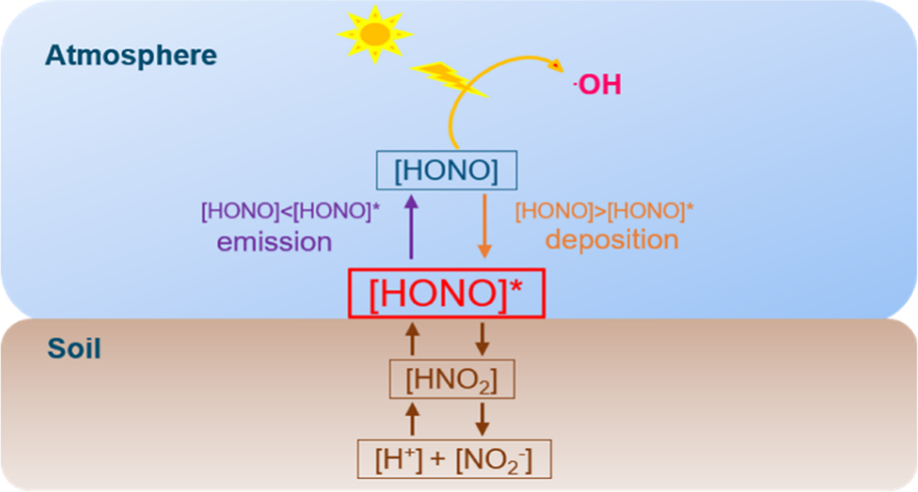

Nitrous acid (HONO) is an important component of the global nitrogen cycle and can regulate the atmospheric oxidative capacity. Soil is an important source of HONO. [HONO]*, the equilibrium gas-phase concentration over the aqueous solution of nitrous acid in the soil, has been suggested as a key parameter for quantifying soil fluxes of HONO. However, [HONO]* has not yet been well-validated and quantified. Here, we present a method to retrieve [HONO]* by conducting controlled dynamic chamber experiments with soil samples applied with different HONO concentrations at the chamber inlet. We show a bi-directional soil–atmosphere exchange of HONO and confirm the existence of [HONO]* over soil: when [HONO]* is higher than the atmospheric HONO concentration, HONO will be released from soil; otherwise, HONO will be deposited. We demonstrate that [HONO]* is a soil characteristic, which is independent of HONO concentrations in the chamber but varies with different soil water contents. We illustrate the robustness of using [HONO]* for quantifying soil fluxes of HONO, whereas the laboratory-determined chamber HONO fluxes can largely deviate from those in the real world for the same soil sample. This work advances the understanding of the soil–atmosphere exchange of HONO and the evaluation of its impact on the atmospheric oxidizing capacity.

## Abstract 2

Equilibrium HONO concentration over soil, [HONO]*, exists as a soil characteristic, which is a key parameter for quantifying soil−atmosphere HONO fluxes.

## Introduction

Hydroxyl radicals (OH) are key species in maintaining photo-oxidation cycles in the atmosphere.^1^ Gaseous nitrous acid (HONO) can be rapidly photolyzed under sunlight to produce OH radicals.^1−4^ In polluted regions, the contribution of HONO to atmospheric OH radical concentrations has been reported to be comparable to or even greater than the contribution of other primary OH sources, for example, the photolysis of ozone and the ozonolysis of alkenes.^5−9^

The main source of atmospheric HONO has been a mystery for decades. Emission from combustion processes^10,11^ and gas-phase production of HONO (via the reaction of NO with OH^12,13^) are not sufficient to explain the observed high atmospheric HONO concentrations in field studies.^14−17^ A heterogeneous reaction of NO_2_ on aerosol surfaces^14,18^ has been suggested to explain the high HONO concentrations.^15,19^ In the presence of light, the reaction has been observed to be significantly enhanced and has been considered to be a missing daytime HONO source.^20,21^ However, the significance of such a source involving NO_2_ uptake on aerosols remains controversial. Under atmospherically relevant conditions, the uptake coefficient of NO_2_ (γ) on aerosols such as mineral dust,^22,23^ soot,^20^ and organic particulates^21^ is at magnitudes of <10^–6^, while γ of >10^–4^ to 10^–5^ is required to explain the observed HONO formation rates of 0.2–2.0 ppb h^–1^.^1,24−27^ Additional HONO formation mechanism such as the photosensitized reduction of NO_2_ on humic acid surfaces has been proposed.^2,28^ Moreover, laboratory studies found that heterogeneous HNO_3_ photolysis on aerosols exhibited a high HONO production rate and has been accounted as an important HONO source.^29−31^ However, multiple scattering effects of light on aerosol sample filters used in those experiments may lead to an overestimation of the observed reaction rates.^32,33^

Besides chemical reactions, Su et al. showed that biogenic soil nitrite can be an important HONO source.^1^ After production from nitrification and denitrification, soil nitrite actively participates in the reversible acid–base reaction [NO_2_^–^ (aq) + H^+^ (aq) ⇔ HNO_2_ (aq)] and releases HONO to the atmosphere through liquid–gas partitioning [HNO_2_ (aq) ⇔ HONO (g)]. Recently, several more studies have been conducted examining soil HONO fluxes^34−42^ and the laboratory chamber fluxes were directly used as estimates of fluxes in the real world.^36,40−42^ One problem of this treatment is the different transfer/deposition velocities of HONO in the laboratory and real-world conditions, which will lead to different fluxes under these two conditions. Up to now, there has been a lack of knowledge on how to translate measured fluxes in the laboratory chamber to those in the real world. In addition, most laboratory measurements have focused on measuring only HONO emission from soil by applying HONO-free air at the chamber inlet.^34,36,39,40^ However, HONO deposition to soil should also be taken into account as it can occur at high atmospheric HONO concentrations. Micrometeorological field measurement methods of HONO fluxes, such as eddy correlation (EC), have been developed to directly determine HONO fluxes in the field.^37,43−45^ However, it is still problematic due to the lack of rapid and sensitive techniques to measure HONO fluxes.^44^ Empirical parameterization and process-based modeling is a labor-efficient alternative and several models have been used to simulate soil HONO fluxes.^34,46−50^ Empirical parameterization models have calculated HONO emissions based on laboratory-determined chamber HONO fluxes as a function of soil water content (SWC).^34,46,51^ A different approach has been suggested by Su et al.^1^ based on the resistance model,^52−54^ as shown in eq 1.
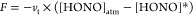
1where [HONO]* is the equilibrium gas-phase concentration over the aqueous solution of nitrous acid [HNO_2_ (aq)] in the soil, [HONO]_atm_ is the atmospheric HONO concentration, and *v*_t_ represents the transfer/deposition velocity of HONO. The resistance model approach, being analogous to electrical current resistance,^54^ accounts for three major processes that limit the transport of HONO from/to soil surfaces including (i) turbulent transport between the atmosphere and the top of the so-called quasi-laminar layer, a very thin layer of stagnant air adjacent to the soil surface, (ii) molecular transport across the quasi-laminar layer, and (iii) emission or deposition from/to the soil surface. Accordingly, three resistances in series, that is, the aerodynamic resistance *R*_a_, the quasi-laminar layer resistance *R*_b,_ and the surface resistance *R*_c_ govern the HONO transport (see Figure S1).^1,55^*v*_t_ equals to the reciprocal of the total resistance (sum of *R*_a_, *R*_b_, and *R*_c_). A major difference between eq 1 and other empirical parameterization methods is that it assumes the existence of [HONO]* and accounts for the effects of transfer/deposition velocities and atmospheric HONO concentrations.^1^ If [HONO]*, as a soil characteristic, indeed exists, we argue that eq 1 is a reliable way to evaluate atmospheric HONO fluxes of soil. However, [HONO]* has not yet been validated and quantified experimentally.

In this study, we aim to demonstrate the existence of [HONO]* over soil, develop a method to derive [HONO]* by controlled dynamic chamber measurements, and quantify the atmospheric soil HONO fluxes. Moreover, [HONO]* variabilities during soil drying processes are investigated.

## Methods

### Sampling

Soil samples were collected on 03 Aug 2020 from an agricultural wheat field in Mainz, Germany (49°59′33.7″N 8°13′05.5″E), at a depth of 0–5 cm. The collected samples were air-dried, grinded, and sieved through a 2 mm cutoff stainless-steel sieve and stored in the dark at room temperature for 3 months before analysis. The physicochemical properties of the soil sample are shown in Table S1.

### Trace Gas-Exchange Measurements

The soil sample was prepared in a Petri glass dish (100 × 20 mm, Duran Group, Germany) containing 50 g of soil and 25 g of pure water (18.2 MΩ). The sample was thoroughly mixed by mechanical stirring and placed in dry purified air at room temperature to reach an SWC of 0.12 kg kg^–1^, corresponding to 31% water holding capacity (WHC) of the soil. The sample was then placed into a dynamic flow-through chamber. The chamber had an inner diameter of 12.0 cm and a height of 13.0 cm. The inner wall material of the chamber was a 50 μm thin transparent Teflon film (FEP) (Saint Gobain Performance Plastics Corporation, USA). To control the temperature of the soil sample, the inner volume of the chamber bottom plate (made of PVDF) was continuously flushed with water cycled using a thermostat (Thermo Fisher Scientific, model SC100). The chamber was purged with purified air derived by passing ambient air through an ozone generator to oxidize nitrogen-containing trace gases, followed by sequential filter columns filled with glass wool (Merck, Germany), silica gel (2–5 mm, Merck, Germany), Purafil (KMnO_4_/Al_2_O_3_, Purafil Inc. USA), and activated charcoal (LS—labor service, Germany). The inlet purging air was humidified using a PID-controlled split (dry/wet) gas system comprising two mass flow controllers (Bronkhorst High-Tech, Netherland) and RH sensors (KFS 140-TO, ±3% accuracy). Downstream of the humidification step, HONO gas was added to the inlet purging air at a small flow rate of 0.02–0.04 mL min^–1^ controlled using another mass flow controller. Many methods have been used to generate stable HONO.^56^ Here, HONO gas was generated by flushing purified air through the headspace of a HONO source solution, which was prepared by dissolving NaNO_2_ (1.25 mM) in a citric acid buffer (pH = 4) solution. The change in the HONO source concentration was less than 0.1 ppb within ∼10 h (Figure S2). From a total airflow of 6.9 L min^–1^, only a fraction of 2.6 L min^–1^ was used to purge the chamber, as this amount was consumed using three gas analyzers. In the overflow exhaust pipe upstream of the chamber, a needle valve was installed. This variable flow resistance was used to keep the inner chamber volume at a slightly higher pressure than ambient, to prevent the risk of laboratory air contaminating the chamber. A Teflon-coated fan was installed in the center of the chamber lid to sustain highly turbulent conditions within the chamber.

HONO was measured with a commercial long path absorption photometer (LOPAP, QUMA, model LOPAP-03, Wuppertal, Germany). The estimated uncertainty of HONO measured by LOPAP is ∼10%. The lower detection limit was calculated from two times the standard deviation of the zero air signal (2σ) at ∼40 ppt for 1 min averages. The LOPAP technique is explained in detail elsewhere.^57^ The gas-phase H_2_O concentration was measured using an infrared CO_2_/H_2_O analyzer operated in the differential mode (LI-7000 LI-COR Biosciences GmbH, Bad Homburg, Germany). The variation of SWC was calculated using the measured differential water vapor concentrations between the chamber inlet and outlet at a given time (*D*_Licor_) and the difference of the mass of the soil (*m*_soil,*t*=0_) prior to and after (*m*_soil,*t*=*N*_) the HONO exchange experiment
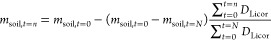
2
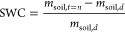
3

Here, *t* = 0 denotes the time when the measurement started, *t* = *N* is the time when the soil dried out, and *t* = *n* is any time between *t* = 0 and *N*. *m*_soil,d_ is the mass of the oven-dried soil, which was determined by putting the soil sample in an oven at 110 °C for 24 h after the HONO exchange experiment. The wall loss of H_2_O and HONO was corrected according to a reference measurement when the chamber was empty. The flow chart of the chamber system is shown in Figure S3.

### [HONO]* Method

In the dynamic flow-through chamber, continuous purging air enters the chamber at the inlet, purges the chamber at a flow rate of *Q*, and exits the chamber at the outlet. The HONO flux (*F*) of soil is related to the transfer/deposition velocity (*v*_t_) of HONO and the gradient between the HONO concentration of the chamber bulk headspace air (*C*_cham_) and the equilibrium HONO concentration over the soil surface, [HONO]*,^1^ here defined as C*
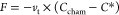
4

To be noted, under ideal conditions, when the equilibrium in the soil is reached, [HONO]* over the soil surface would be the same as that in the soil. In practice, the equilibrium is expected to be reached within a shallow topsoil layer.

The HONO flux (*F*) can also be quantified using the chamber mass balance equation^58^
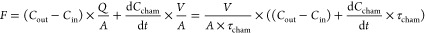
5

Here, *V* is the chamber volume and *A* denotes the soil surface area, *C*_in_ and *C*_out_ are the HONO concentrations measured at the chamber inlet and outlet, respectively, τ_cham_ is the residence time () of the air within the chamber volume, and t is the experiment time. During the experiment, HONO concentrations at the chamber inlet were switched between three concentrations at time intervals of 15 min. The measured HONO concentrations from only minute 11 to 13 of each time interval were used to calculate [HONO]*.  in this time duration was negligible (0.02 ± 0.23 ppb), compared to *C*_cham_ (13.9 ± 8.13 ppb). Therefore, eq 5 can be reduced to
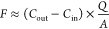
6

To be noted, eq 6 is only valid for calculating the flux of an inert trace gas, which shows no reactions with other air components in the chamber. HONO is chemically reactive under UV light. Since this study was conducted in the dark, HONO was considered inert. In addition, HONO concentration of the chamber headspace (*C*_cham_) can be assumed uniform as the chamber air was well-mixed and hence also equals to the concentration measured at the chamber outlet (*C*_out_)
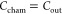
7

Combining eqs 4, 6, and 7 gives
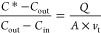
8

When applying *C*_in1_ of HONO concentration at the chamber inlet and measuring the concentration at the chamber outlet (*C*_out1_), the following equation is obtained
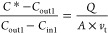
9

When HONO concentration at the chamber inlet was switched to *C*_in2_, the concentration at the chamber outlet (*C*_out2_) was measured. A similar equation as eq 9 is obtained
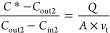
10

Here, soil conditions during *C*_out2_ were the same as during the determination of *C*_out1_ (see Figure S4 for details), and thus, parameters on the right-hand side of eqs 9 and 10 are the same and can be combined as
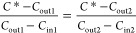
11

In this way, the unknown parameters (*A* and *v*_t_) are canceled out and C* can be solved from eq 11
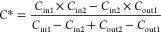
12

The method is applicable to obtain equilibrium concentrations not only of HONO but also of other trace gases over soil and other surfaces. Because the equilibrium relative humidity (RH*) of air over a liquid water surface is known to be 100%, a chamber test with liquid water in a Petri dish was performed to validate the applicability of the abovedescribed C* method by comparing the observation-based RH* with the theoretically assumed 100% (see the Supporting Information for details). The RH* results showed no dependence on different chamber turbulent conditions (Figure S5 and Table S2) and the mean of the RH* results of all tests was 97.4%. The consistency of mean RH* under different chamber turbulent conditions and the proximity of the RH* to 100% confirm the validity of the C* method, that is, retrieving C* from a set of two different inlet concentrations while monitoring the respective outlet concentrations.

## Results and Discussion

### HONO Exchange of Soil at Different Inlet HONO Concentrations

**Figure 1:**
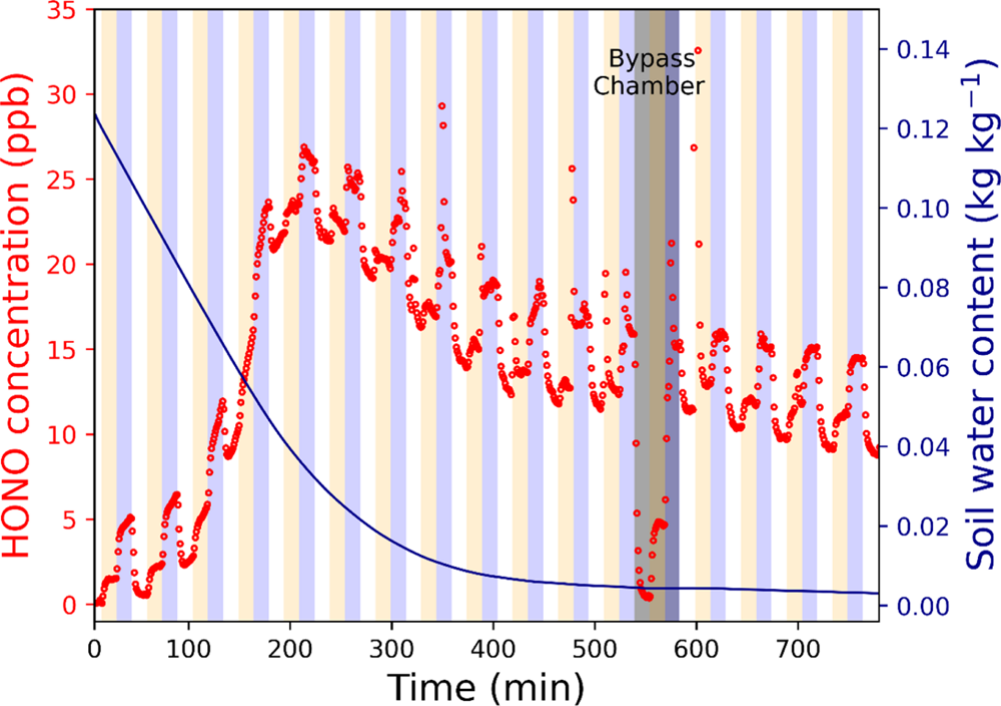
Change in HONO concentration at the chamber outlet (left, red) and SWC (right, dark blue) over time of the soil drying process when the HONO concentration of the inlet purging air was switched between 0 ppb (white-shaded), 5 ppb (yellow-shaded), and 15 ppb (blue-shaded) in 15 min intervals. The grey-shaded area indicates when the inlet purging air bypassed the chamber to check the stability of the inlet HONO concentrations.

Figure 1 shows the results of a HONO exchange experiment of a soil sample. The SWC gradually decreased during the experiment as semihumidified air (46% RH) was applied. Inlet HONO concentrations were sequentially switched between three different concentrations (0, 5, and 15 ppb) in 15 min intervals. For all three inlet HONO concentrations, the HONO concentration at the chamber outlet exhibited a similar trend with respect to the decreasing SWC. Observed outlet HONO concentrations first increased as the SWC decreased. After reaching a maximum at an SWC of 0.04 kg kg^–1^ (10% WHC), the HONO concentrations decreased. This pattern of soil HONO emission during the soil drying process is similar to that found in previous studies.^1,36^

**Figure 2:**
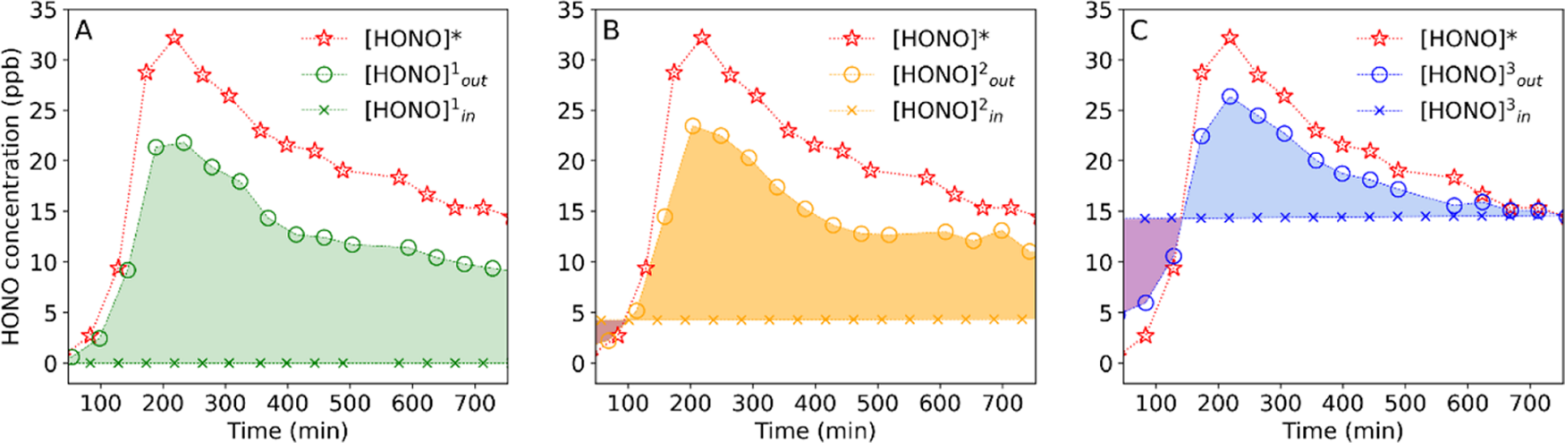
HONO concentration (O) at the chamber outlet when the inlet HONO concentration (X) was switched between 0 (A), 5 (B), and 15 ppb (C) and the equilibrium HONO concentration ([HONO]* (☆), the mean of [HONO]*_1_ and [HONO]*_2_, see Figure 3) over time of the soil drying process. Color-shaded areas indicate periods of emission (light color) or deposition (dark color), respectively. Note that Figure 2 was obtained by separating the data in Figure 1 according to the three inlet HONO concentrations applied.

To see the influence of inlet HONO concentrations on the soil HONO fluxes, the outlet HONO concentration data were evaluated independently from each other according to the three inlet HONO concentrations applied (Figure 2). At an inlet HONO concentration of 0 ppb, net emission of HONO persisted throughout the whole soil drying process. At 5 and 15 ppb of HONO applied at the inlet, the outlet HONO concentration was first found lower than the inlet HONO concentration, indicating net HONO deposition to the soil. As the SWC continued to decrease, net HONO emission was observed. These results show that either HONO emission from or deposition to soil occurs at different inlet HONO concentrations. Different inlet HONO concentrations cause different HONO concentrations in the chamber headspace, which correspond to atmospheric concentrations of HONO in the real world. These results suggest that whether HONO is emitted from or deposited to soil depends not only on soil properties but also on atmospheric HONO concentrations, contributed by different HONO sources and sinks.

**Figure 3:**
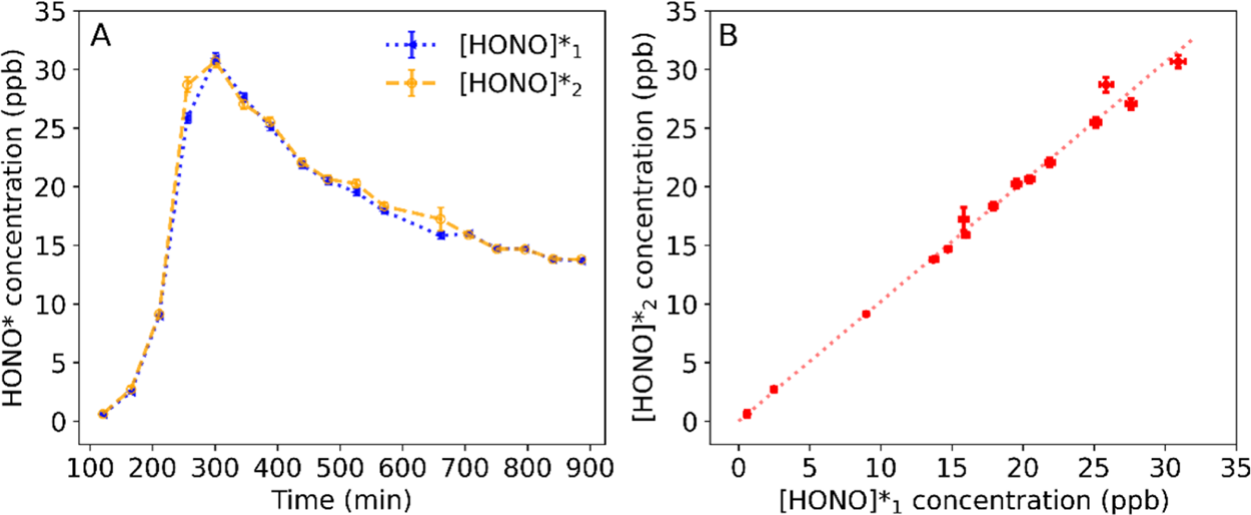
Equilibrium HONO concentrations, [HONO]*_1_ (blue) and [HONO]*_2_ (orange) over time of the soil drying process (A) and their correlation with each other (B). The red dotted line represents the linear fit on the data points (slope = 1.02, *R*^2^ = 1.00). [HONO]*_1_, [HONO]*_2_, and [HONO]*_3_ (Figure S6) were calculated based on three different result combinations of the applied inlet HONO concentrations, that is, grouping 0 ppb and 15 ppb, grouping 5 and 15 ppb, and grouping 0 and 5 ppb, respectively. Error bars indicate the uncertainties in the [HONO]* retrieval, estimated by the Monte Carlo method (see details in Figure S6). Note that the uncertainty of [HONO]*_3_ was large with an average of ±1.02 ppb, compared to that of [HONO]*_1_ (±0.27 ppb) and [HONO]*_2_ (±0.34 ppb). Therefore, [HONO]*_3_ was not included here for comparison.

**Figure 4:**
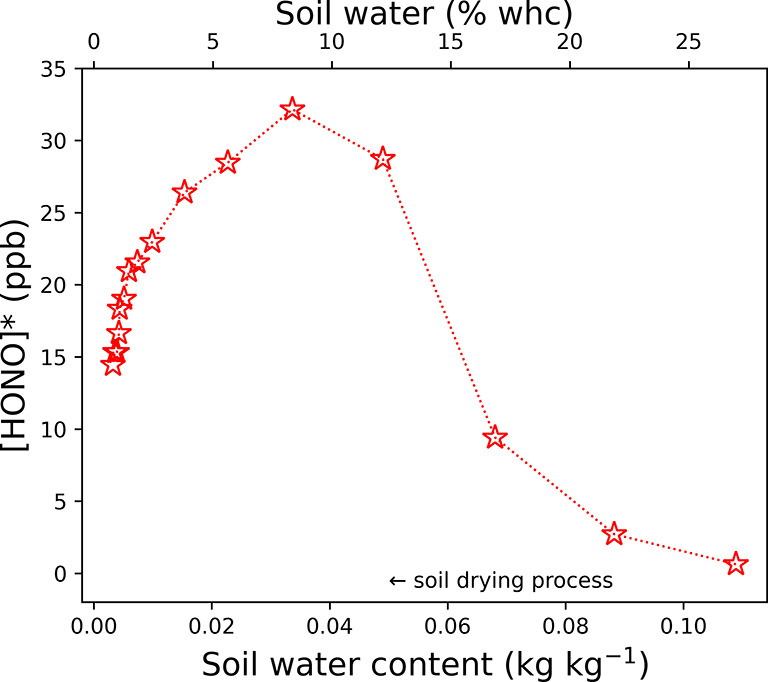
Equilibrium HONO concentrations [HONO]* (mean of [HONO]*_1_ and [HONO]*_2_) at different SWCs during the soil drying process.

[HONO]*, the equilibrium gas-phase HONO concentration over the soil, has been suggested to be an important parameter to determine the bi-directional HONO exchange between soil and the atmosphere.^1^ Up to now, only theoretical [HONO]* values have been calculated according to pH and nitrite content of bulk soils.^1,59^ In this work, we introduced a method to retrieve the actual [HONO]* values during the soil drying process (see the Methods for details). To check for consistency, two different result combinations of applied inlet HONO concentrations were used to calculate [HONO]* according to eq 12, that is, grouping 0 and 15 ppb ([HONO]*_1_) and grouping 5 and 15 ppb ([HONO]*_2_). As shown in Figure 3, there was a close correlation between [HONO]*_1_ and [HONO]*_2_. These results show that the retrieved [HONO]* is independent of the inlet HONO concentrations applied, which proves that [HONO]* indeed exists as a soil characteristic. As shown in Figure 2, [HONO]* regulates both the direction and the magnitude of HONO exchanges from/to the soil. When [HONO]* is higher than the HONO concentration of the chamber headspace air ([HONO]_out_), HONO will be released from the soil; otherwise, HONO will be deposited to the soil. In the real world, the comparison between [HONO]* and atmospheric HONO concentrations can predict whether HONO is emitted from or deposited to the soil. Furthermore, [HONO]* is strongly dependent on SWC as shown in Figure 4. As the SWC decreased, [HONO]* increased to a maximum (∼31 ppb) at an SWC of 0.04 kg kg^–1^ (10% WHC) and then [HONO]* decreased as SWC further decreased. When SWC decreases, the increasing concentration of HNO_2_ (aq) in soil water can lead to a higher [HONO]*, according to Henry’s law behavior of gas–liquid partitioning [HNO_2_ (aq) ⇔ HONO (g)]. This, however, could not explain the decrease in [HONO]* when SWC further decreases. A possible explanation is the limited kinetic mass transport and the nonideal solution behavior at lower SWC.^60^

### Quantification of Soil HONO Fluxes

**Figure 5:**
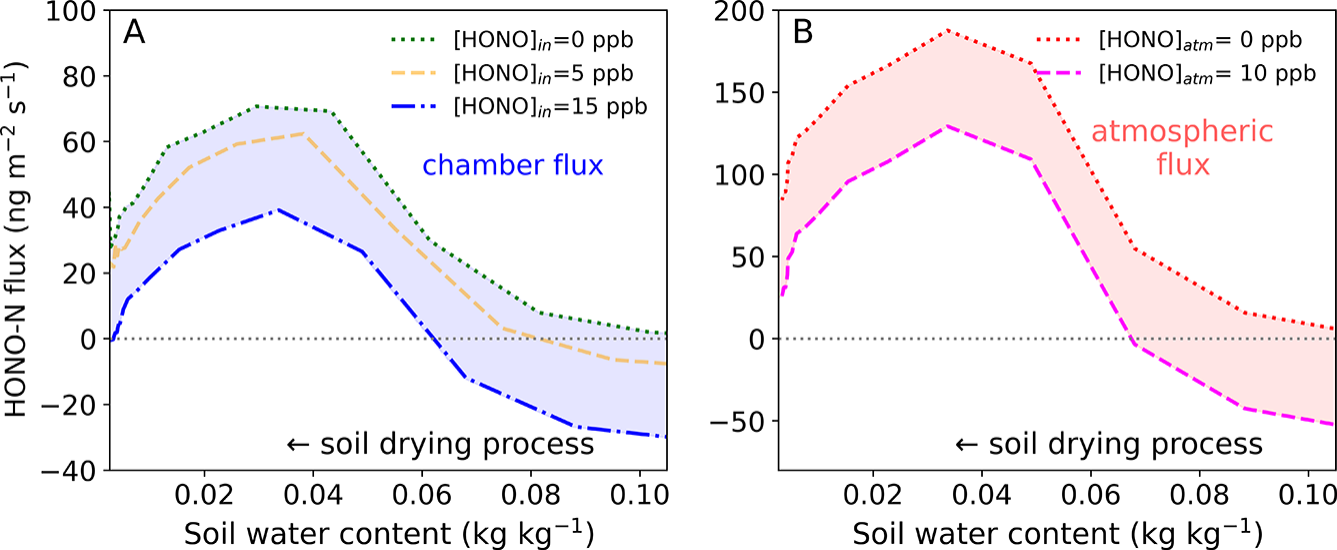
Chamber HONO fluxes (A) calculated by eq 6 on the basis of soil geometric surface area when inlet HONO concentrations were 0 ppb (···, green), 5 ppb (---, orange), and 15 ppb (-·-, blue) and atmospheric HONO fluxes (B) calculated by eq 1 using equilibrium HONO concentrations ([HONO]*) at different SWCs during the soil drying process retrieved by the [HONO]* method. Atmospheric HONO concentrations ([HONO]_atm_) of 0 (···, pink) and 10 ppb (---, magenta), both with a HONO transfer/deposition velocity (*v*_t_) of 1 cm s^–1^ were adopted for calculation.

The chamber HONO flux from/to the soil sample was calculated according to eq 6. At the three inlet HONO concentrations, the chamber HONO fluxes ranged from −31.1 to 68.6 ng N m^–2^ s^–1^ at different SWCs during the soil drying process (Figure 5A). In previous studies, HONO fluxes of soil derived from dynamic chamber measurements have been adopted directly to predict fluxes in the real world.^36,40−42^ However, fluxes determined in the laboratory chamber can vary greatly from fluxes in the real world, mainly due to the different transfer/deposition velocities under laboratory conditions and real-world conditions. We show this by examining chamber H_2_O vapor fluxes of pure liquid water as a well-controllable proxy for fluxes of trace gas species (Figure S7). Chamber H_2_O vapor fluxes were observed to be strongly dependent on chamber turbulent conditions. On the other hand, as noted above in the [HONO]* method section, the turbulent conditions did not significantly affect the observation-based RH* (Figure S5), which indirectly demonstrates the robustness of the [HONO]* method.

[HONO]* is a soil characteristic and does not depend on chamber mixing levels or purging flow rates, thus being better suited to estimate HONO fluxes in the real world. Soils from different environments can be collected and [HONO]* can be retrieved by the described [HONO]* method. In addition to [HONO]*, the transfer/deposition velocity (*v*_t_) and the atmospheric concentration of HONO ([HONO]_atm_) are also crucial parameters for quantifying soil HONO fluxes according to eq 1. *v*_t_ depends primarily on the meteorological conditions and soil resistances, and the reported *v*_t_ values fall in the range of 0.077–3 cm s^–1^.^61−65^ Field observations of [HONO]_atm_ have been extensively performed (Table S3) and up to ∼10 ppb of [HONO]_atm_ have been reported for a fertilized agricultural field site in the North China Plain.^66^ [HONO]* at different SWCs during the drying process of an agricultural soil sample was determined by the present study (Figure 4). Accordingly, the predicted atmospheric HONO fluxes range from −54.8 to 179.6 ng m^–2^ s^–1^ if adopting [HONO]_atm_ of 0–10 ppb and a typical *v*_t_ of 1 cm s^–1^ (Figure 5B).^1^ When adopting *v*_t_ of 0.077–3 cm s^–1^, the predicted atmospheric HONO fluxes range from −164.5 (when [HONO]_atm_ = 10 ppb) to 538.7 ng m^–2^ s^–1^ (when [HONO]_atm_ = 0 ppb), as shown in Figure S8. These results show that the predicted atmospheric HONO fluxes can differ widely from HONO fluxes measured in the chamber (Figure 5A), which is due to different HONO concentrations and *v*_t_ in the chamber and in the real world. These results illustrate that the chamber-derived HONO fluxes cannot be directly used to estimate HONO fluxes of soil in the real world.

Meusel et al. estimated [HONO]* by a simplified method using measurements of the water–air exchange of H_2_O vapor.^39^ Assuming a similar concentration gradient between the chamber headspace air and the surfaces of soil and liquid water for HONO and H_2_O vapor, [HONO]* can be estimated by
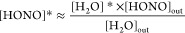
13where [H_2_O]* is the known saturation water vapor concentration (100% RH), [H_2_O]_out_ is the measured H_2_O concentration at the chamber outlet with inlet dry air, and [HONO]_out_ is the measured HONO concentration at the chamber outlet at an inlet HONO concentration of 0 ppb. In Figure S9, [HONO]* derived by the [H_2_O] method shows a good agreement (within 30%) with that derived by the [HONO]* method of the present study. This suggests that the transfer/deposition velocities, *v*_t_, of HONO and H_2_O vapor are similar in the same chamber system. *v*_t_ equals to the reciprocal of the sum of *R*_a_, *R*_b_, and *R*_c_ (Figure S1). *R*_a_, the aerodynamic resistance, is the same for surface–air exchange of these two species in the same chamber system. Smaller [HONO]* values derived by the [H_2_O] method than those by the [HONO]* method could be explained by the smaller *R*_b_, quasi-laminar layer resistance, and *R*_c_, surface resistance for the water–air exchange of H_2_O vapor than those for soil–air exchange of HONO. For the [HONO]* method, two sets of HONO measurement results under the same soil and chamber conditions were used for [HONO]* calculation, which guaranteed that *R*_a_, *R*_b_, and *R*_c_ were all the same. [HONO]* can then be solved accurately.

As aforementioned, theoretical [HONO]* can be calculated from values of nitrite concentration and pH in soil water, along with temperature and SWC.^1^ Assuming an SWC of 0.04 kg kg^–1^, the theoretical [HONO]* of the soil sample at the experiment temperature (22 °C) was calculated to be ∼0.3 ppb according to its nitrite content (0.43 mg kg^–1^) and pH (7.7) measured before the soil drying process. In comparison, the observation-based [HONO]* at an SWC of 0.04 kg kg^–1^ was ∼30.8 ppb (Figure 4). The deviations can be caused by a variable nitrite content during the soil drying process due to active N-transforming microorganisms.^67^ In addition, the nitrite concentrations and pH across the soil can vary by orders of magnitude.^48^ Besides the dynamics, surface layer soil or soil solution is also not an ideal solution, the nonideality and adsorption equilibrium may differ from the results based on an ideal solution system. Moreover, the kinetic limitation, for example, change in diffusion in soil water pores due to restricting of soil water in the course of drying, would also play a role in the change in HONO fluxes, which would further complicate the [HONO]* calculation. In contrast, the [HONO]* method in the present study determines an overall equilibrium concentration over the soil surface. Therefore, the observation-based [HONO]* values (Figure 4) are more atmospherically relevant in quantifying soil–atmosphere HONO fluxes.

### Atmospheric Implication

The present study shows that the exchange of HONO between soil and the atmosphere is bi-directional and provides direct evidence of the [HONO]* existence over soil. This soil–atmosphere exchange of HONO is mainly regulated by [HONO]* and the atmospheric HONO concentration ([HONO]_atm_). Both [HONO]* and [HONO]_atm_ can be affected by various environmental factors. For example, high temperature during daytime leads to increased [HONO]* due to the temperature dependence of the equilibrium of HNO_2_ dissociation and gas–liquid partitioning on the soil surface.^1^ At the same time, the photolysis of HONO under sunlight causes a relatively low daytime [HONO]_atm_. As a result, [HONO]* is larger than [HONO]_atm_ during daytime and HONO emission from the soil should prevail. In this scenario, soil will be a daytime HONO source, which helps us to explain the missing HONO sources observed during daytime.^5,68^ During nighttime, a decreased [HONO]* at low temperature and a relatively high [HONO]_atm_ due to the absence of photolysis can lead to HONO deposition to the soil and soil will be a HONO sink. Therefore, such diurnal variations of temperature and sunlight could lead to diurnal patterns of HONO fluxes between soil and the atmosphere. Our predicted pattern is in accordance with the observed diurnal HONO fluxes in an agricultural field.^66^ Besides temperature, other parameters can also influence [HONO]*, such as SWC, soil nitrite, pH, and microbial activity. Although there is an increasing body of field measurements of [HONO]_atm_, experimental investigations and model simulations on [HONO]* are still required to unravel and quantify soil HONO fluxes under different environmental conditions. In addition, [HONO]* is also linked with soil moisture and chemical, physical, and biological processes in the soil. We recommend further studies to investigate the dependence of [HONO]* on HONO gas–liquid/gas–solid exchanges and the kinetic mass transport in the soil. It is also feasible to apply the [HONO]* method in the field to derive [HONO]* of soil with its original properties and thickness. Investigations of [HONO]* could improve our predictions of atmospheric HONO fluxes of soil and our understanding of how the biosphere affects air quality and global climate.

## Supporting Information Available

Details of validation of the equilibrium relative humidity (RH*) over a pure liquid water surface, resistance model for HONO fluxes of soil, stability of inlet HONO concentrations, chamber system, interpolation of *C*_out_ for [HONO]* calculation, RH* measurements, HONO* uncertainty, chamber H_2_O vapor fluxes under different chamber conditions, predicted atmospheric HONO fluxes when adopting different [HONO]_atm_ and *v*_t_, comparison of [HONO]* derived by two methods, soil physicochemical properties, RH* under different chamber turbulence conditions, and summarizing HONO measurements (PDF)
