# Supplementary material for: Key Role of Equilibrium HONO Concentration over Soil in Quantifying Soil–Atmosphere HONO Fluxes
Source: Environ Sci Technol. 2022 Feb 1;56(4):2204–12. doi: 10.1021/acs.est.1c06716 (PMC8851686; doi:10.1021/acs.est.1c06716)
Supplement: Supplementary file 1 — es1c06716_si_001.pdf [file es1c06716_si_001.pdf]

Supporting Information

Key Role of Equilibrium HONO Concentration over Soil in Quantifying Soil-Atmosphere HONO Fluxes

Fengxia Bao[1], Yafang Cheng[1, 2], Uwe Kuhn[1], Guo Li[1], Wenjie Wang[1], Alexandra Maria Kratz[1], Jens Weber[3,1], Bettina Weber[3,1], Ulrich Pöschl[1], Hang Su[1*]

[1] Max Planck Institute for Chemistry, Mainz, Germany

[2] Department of Precision Machinery and Precision Instrumentation, University of Science and Technology of China, Hefei, China

[3] Institute of Biology, University of Graz, Graz, Austria

*Corresponding author:

Dr. Hang Su E-mail: h.su@mpic.de

Number of pages: 12

Number of Figures: 9

Number of Tables: 3

Validation of the equilibrium relative humidity (RH*) over a pure liquid water surface: We applied pure liquid water in a Petri dish in the described chamber system continuously flushed with dry/humidified N_2. The H_2O vapor concentration of the purging air bypassing the chamber was measured as C_{in}. The H_2O vapor concentration of the purging air inside the chamber was measured as C_{out}. According to eq 12 in the main text, two sets of measured H_2O concentrations C_{out} and C_{in} allow the determination of C*. Thus, RH* can be calculated from C* according to:

$$\mathrm{RH}^* = \frac{C^*}{C_{sat}} \qquad (1)$$

In the above eq, C_{sat} is the H_2O vapor saturation mole fraction (in mmol mol^{-1}). As C_{sat} is strongly dependent on temperature, the temperature of the H_2O liquid surface was measured by a DS18B20 digital thermosensor (maxim integrated, U.S., accuracy of ± 0.5°C, from -10°C to + 85°C) located close to the water surface.

Table S2 summarizes the RH* results of the liquid H_2O sample under different chamber turbulence conditions, which were applied by using different flow rates (3 - 4 L min^{-1}) and fan speeds (voltage 2 - 3 V). For each set of turbulent conditions, the H_2O vapor concentration at the chamber outlet was monitored, while sequentially applying two different water vapor concentrations at the chamber inlet (C_{in1} and C_{in2}). C_{in1} was fixed as 0 % and C_{in2} was increasing from 20% to 90% RH over a fixed time-span of 30 minutes. Wall loss and artifacts were corrected by means of reference measurements when the chamber was empty. RH* was calculated using the corrected results.

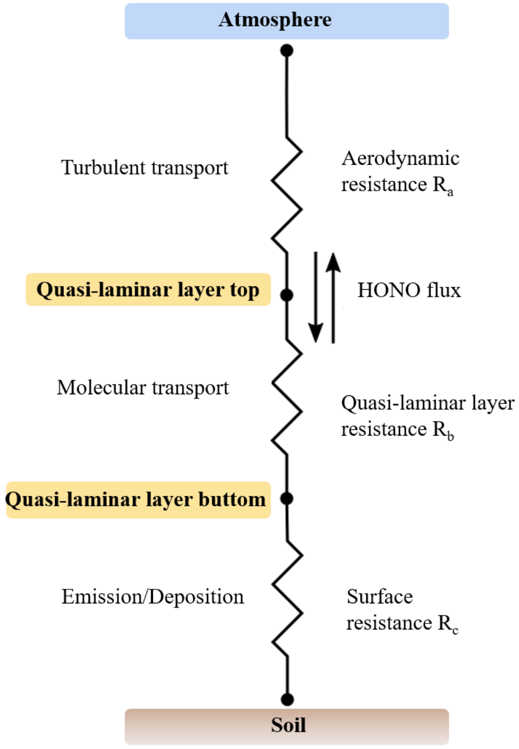

Figure S1. Resistance model[1] for HONO fluxes of soil.

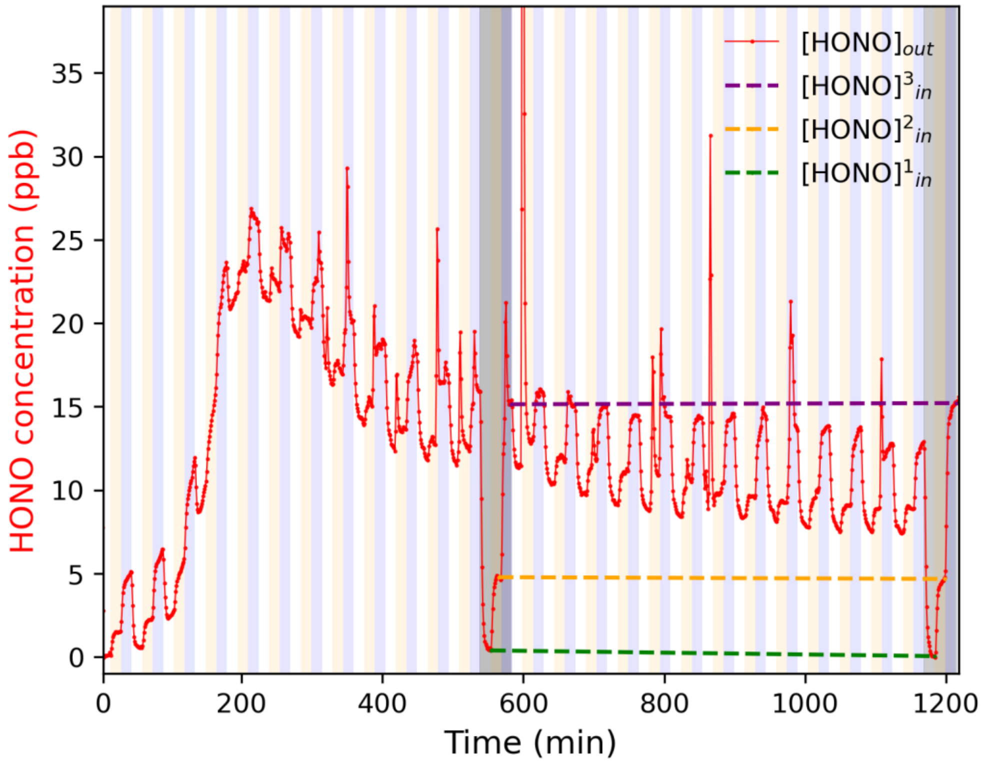

Figure S2. Change in HONO concentration at the chamber outlet over time of the soil drying process when inlet HONO concentration was switched between 0 ppb (white-shaded), 5 ppb (yellow-shaded), and 15 ppb (blue-shaded) in 15-minute intervals. The grey-shaded area

indicates when the inlet purging air bypassed the chamber to check the stability of the inlet HONO concentrations (green (5 ppb), orange (10 ppb) and purple (15 ppb) dashed lines). Only the first 800 min of data are shown in the main article for clarity.

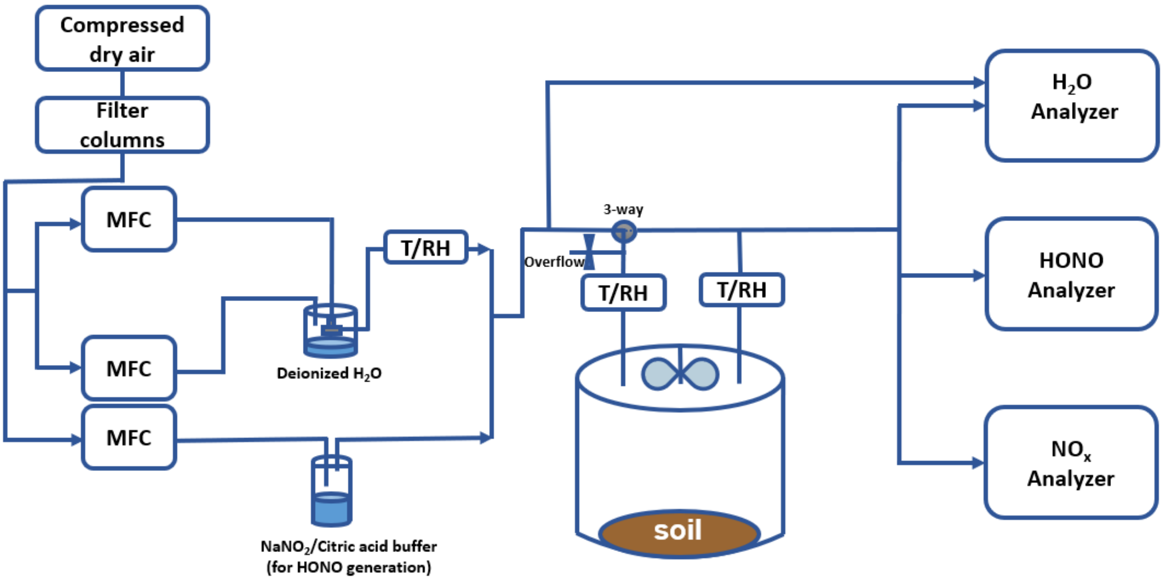

Figure S3. Flow chart of the chamber system

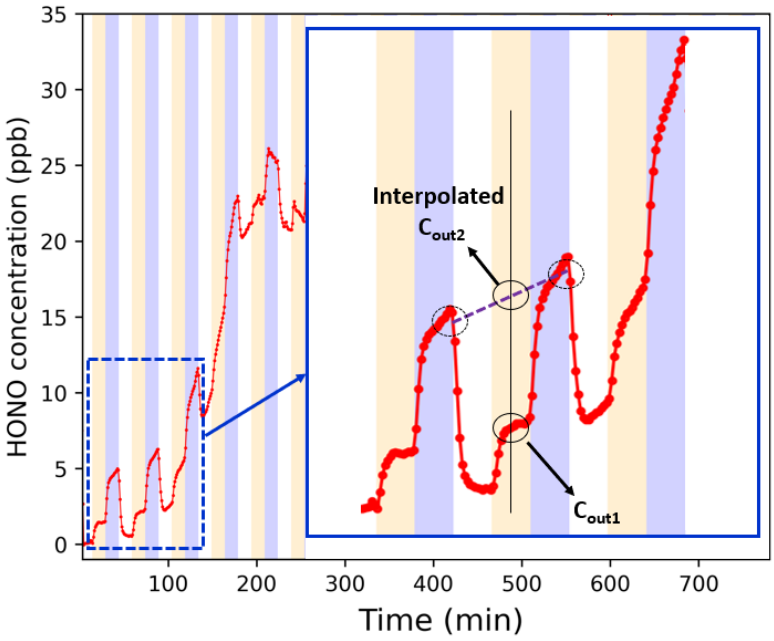

Figure S4. Change in HONO concentration at the chamber outlet over time of the soil drying process when the HONO concentration of the inlet purging air was switched between 0 ppb (white-shaded), 5 ppb (yellow-shaded), and 15 ppb (blue-shaded) in 15-minute intervals (same as in Figure 1 of the main text). The inset figure shows the interpolation of C_{out} for [HONO]* calculation.

After switching the HONO concentration at the chamber inlet from C_{in1} to C_{in2}, the concentration C_{out2} at the chamber outlet was measured. To be able to reflect the same soil conditions for the C_{out2} measurements as apparent during the C_{out1} measurements, the two C_{out2} concentrations directly before and after C_{out1} were interpolated to derive a virtual C_{out2} representative for the time period when C_{out1} had been measured. In this way, the prerequisite that the two measurements and parameters in eqs 9 and 10 in the main text are the same is justified.

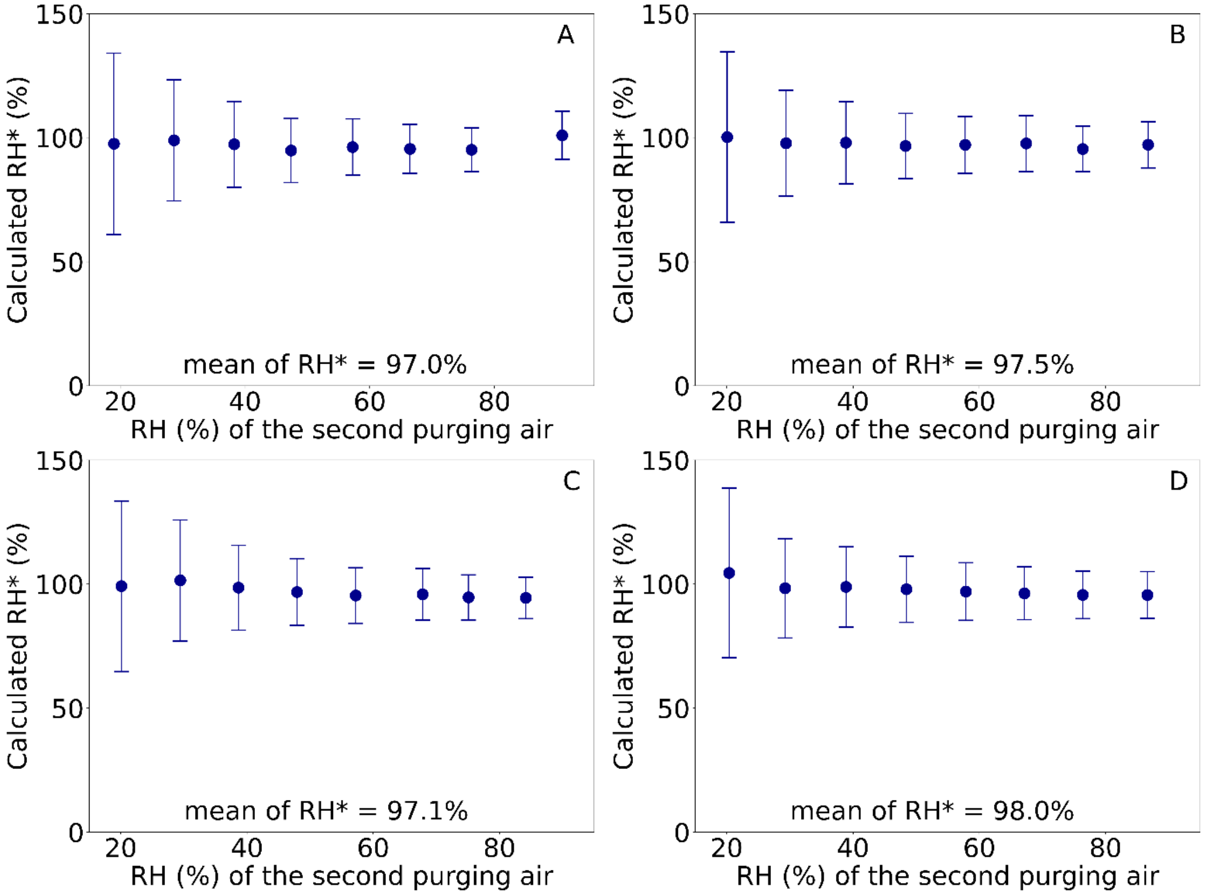

Figure S5. RH*, the equilibrium relative humidity (RH) over a H_2O liquid surface in different chamber turbulence conditions (A) at a fan voltage of 3 V and purging flow rate of 3 L min^{-1}; (B) at a fan voltage of 3 V and purging flow rate of 4 L min^{-1}; (C) at a fan voltage of 2 V and purging flow rate of 3 L min^{-1}; (D) at a fan voltage of 2 V and purging flow rate of 4 L min^{-1} calculated according to the combined results applying two different RH (%) of the purging air at the chamber inlet. The first inlet RH was fixed to 0% and the second was changing from 20% to 90% RH. Error bars indicate the uncertainties of the RH* retrieval, estimated using the Monte Carlo method, as detailed below.

The uncertainty of the derived RH* could be caused by the uncertainty of input parameters, i.e.

±3% for H_2O vapor concentration and ± 0.5 °C for temperature, which was estimated using the Monte Carlo method. For each input parameter in the RH* calculation, 40,000 Latin-hypercube samples were used, which were sufficient for the convergence of the mean and standard deviation (*std*). The samples were normally distributed around the original input value with 1*std*. 3*std* of the calculation results of RH* from the Latin hypercube samples were considered as uncertainty (at a confidence level of 99%). As shown in Figure S4, a larger uncertainty occurred when C_{in1} and C_{in2} were closer. Therefore, a large enough difference between C_{in1} and C_{in2} should be given to ensure low uncertainties. The deviation between the observation-based RH* of 97.4% and 100% RH is well within the uncertainty retrieved by the Monte Carlo method.

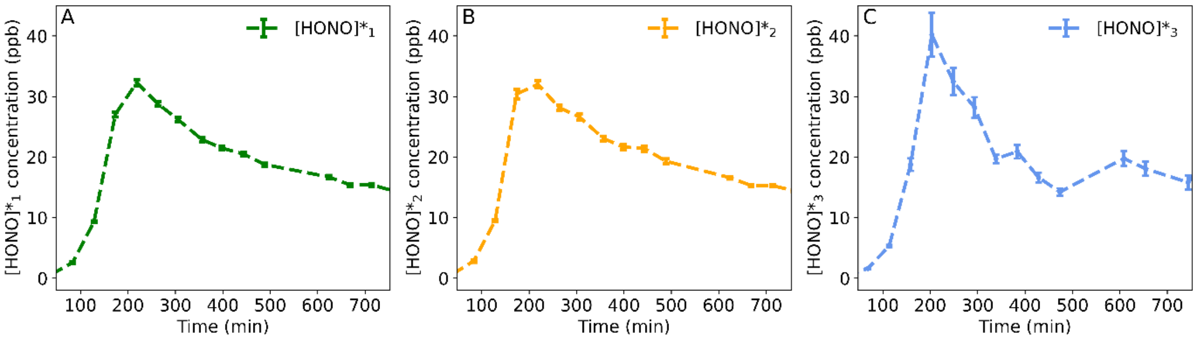

Figure S6. Equilibrium HONO concentrations $[HONO]^*_1$ (A), $[HONO]^*_2$ (B), and $[HONO]^*_3$ (C) over time of the soil drying process. $[HONO]^*_1$, $[HONO]^*_2$ and $[HONO]^*_3$ were calculated based on three different result combinations of the applied inlet HONO concentrations, i.e., grouping 0 ppb and 15 ppb, grouping 5 ppb and 15 ppb, and grouping 0 ppb and 5 ppb, respectively. Error bars indicate the uncertainties in the [HONO]* retrieval, estimated using the Monte Carlo method, described in detail below.

Similar to the estimation of RH* uncertainty (Figure S5), the uncertainty of the derived HONO* was also estimated using the Monte Carlo method. 40,000 Latin-hypercube samples were used for each input parameter in eq 12 for HONO* calculation. The samples were normally distributed around the original input value with 1*std* (40 ppt). 3*std* around the computational results of HONO* were considered as overall uncertainty. The uncertainty of $[HONO]^*_3$ was much larger compared to that of $[HONO]^*_1$ and $[HONO]^*_2$. As aforementioned (Figure S5), a larger uncertainty occurs when two applied inlet concentrations are too close to each other and thus this should be avoided.

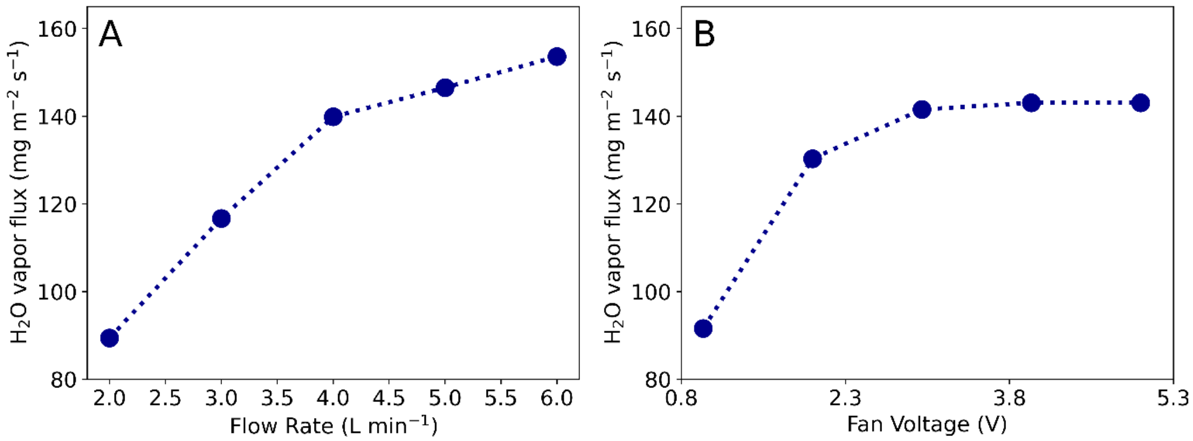

Figure S7. Chamber H_2O vapor fluxes as a function of the flow rate of the purging air at a fixed mixing fan speed (voltage of 4 V) (A) and as a function of the chamber fan voltage at a fixed purging air flow rate of 4 L min^{-1} (B).

10 ml of liquid phase H_2O in a Petri dish (100 x 20 mm) was placed into the dynamic chamber, which was continuously purged with dry purified air. Due to H_2O evaporation in the chamber, the purging air was humidified and the H_2O vapor concentration was measured at the chamber outlet. The H_2O vapor fluxes were calculated when a steady-state had developed in the chamber. Different sets of chamber turbulent conditions were adjusted by changing the flow rate of the purging air and the spinning speed of the chamber fan. Chamber H_2O vapor fluxes were observed to increase with flow rates of the purging air. Moreover, the spinning speed of the chamber fan can also influence the H_2O vapor flux. The impact of chamber flow rates and turbulence on chamber fluxes revealed that there are significant uncertainties when simply using the measured chamber fluxes as estimates of the fluxes in the real world.

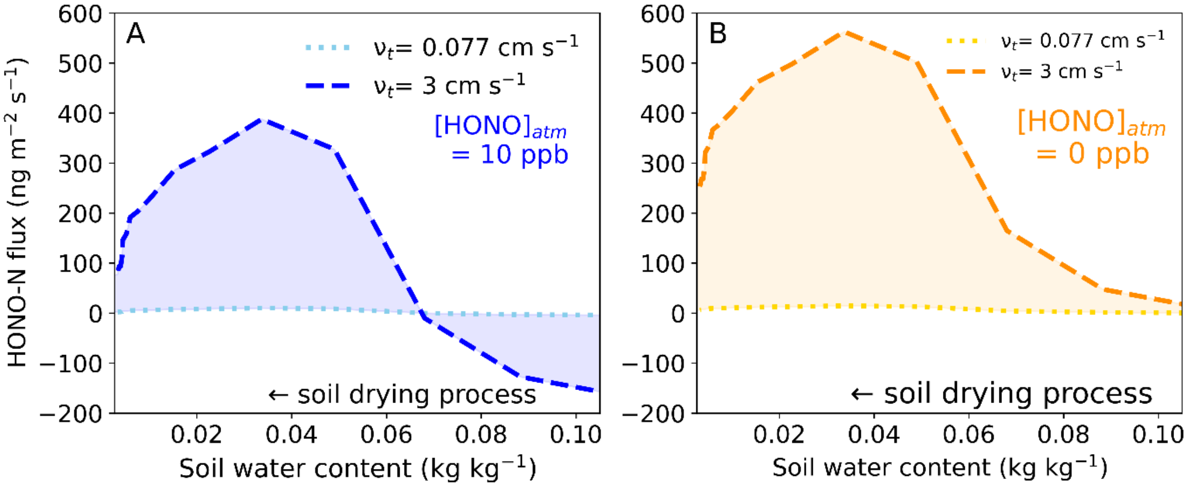

Figure S8. Predicted atmospheric HONO fluxes when adopting equilibrium HONO

concentrations ([HONO]*) at different SWC during the soil drying process obtained in this study, HONO transfer velocities (v_t) of 0.077 (···, light color) and 3 cm s^{-1} (---, dark color) and atmospheric HONO concentrations $[HONO]_{atm}$ of 10 ppb (A) and 0 ppb (B).

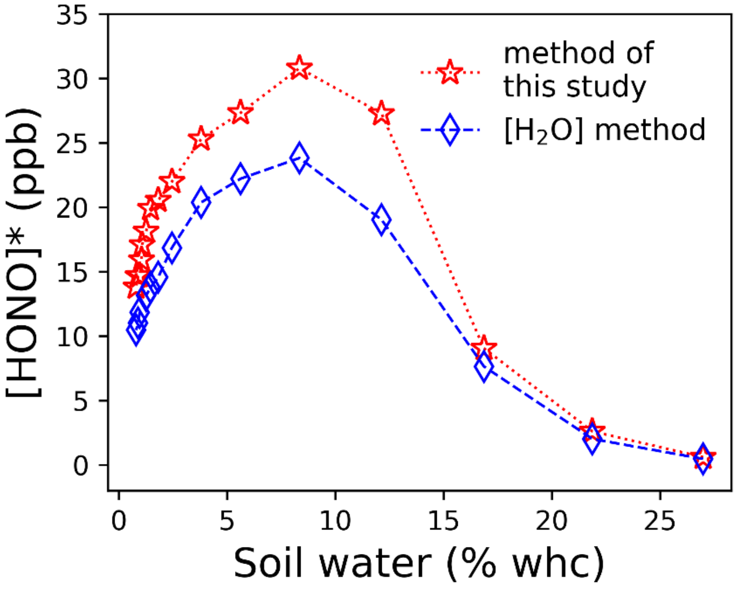

Figure S9. Equilibrium HONO concentrations [HONO]* derived by the method of the present study (☆, red) and by the $[H_2O]$ method (◊, blue) based on $[HONO]^1_{out}$ results in Figure 2a at different soil water contents during the soil drying process.

Table S1. Physicochemical soil properties

nitrite mg kg^{-1}	nitrate mg kg^{-1}	ammonium mg kg^{-1}	pH 1	sand %	clay %	silt %
0.43	68.86	<DL	7.7	43.52	51.40	5.08

Nutrient contents are shown in terms of N; Detection limit (DL) of ammonium is 0.5 mg kg^{-1}. The physicochemical properties were determined by Envilytix GmbH (Wiesbaden, Germany). Nitrite, nitrate and ammonium of the soil (1:5 water) were determined by photometry. The pH of soil (1:5 water) was determined according to DIN ISO 10390 and soil texture was determined according to DIN ISO 11277.

Table S2. RH*, the calculated equilibrium relative humidity over a liquid water surface under different chamber turbulent conditions

Flow rate	Fan voltage	Mean RH*

3	2	97.1
4	2	98.0
3	3	97.0
4	3	97.5

Table S3. Summary of HONO measurements

Location	Date	HONO (ppb)	References
Beijing/China	Nov 2018-Jan 2019	0.02-3.18	2
Melpitz /Germany	Apr 2018	0.10-0.40	3
Baoding/China	Dec 2017	1.80	4
Dongying/China	Feb-Mar 2017	0.26	5
Baoding/China	June 2017	4.36-9.49	6
Beijing/China	Dec 2016	0.18-9.00	7
Seoul/Korea	May-Jun 2016	0.04-3.46	8
Tokyo/ Japan	Jan- Feb 2016	1.50	9
Jinan/ China	Dec 2015-Feb 2016	1.70	10
Beijing/China	Sep 2015-Jul 2016	1.40	11
North Atlantic	Nov-Dec 2015	0.01	12
Xi’an/China	July-Aug 2015	0.02-4.30	13
London/ UK	Jul-Aug 2012	0.20-1.80	14
Erie/USA	Feb-Mar 2011	0.10	15
Boreal forest/Finland	Jul-Aug 2010	0.03	16
Paris/France	Jan- Feb 2010	0.40-0.50	17
Barrow/Alaska	Mar-Apr 2009	0.03	18
Beijing/China	Feb 2007	0.15-9.71	19
Seoul/Korea	May-July 2005	0.36	20
Xinken/China	2004	0.4-3.5	21

REFERENCES

(1) Seinfeld, J. H.; Pandis, S. N. *Atmospheric Chemistry and Physics: From Air Pollution to Climate Change*, 3rd ed.; Wiley: New York, 2016. https://doi.org/10.1128/AAC.03728-14.

(2) Liu, J.; Liu, Z.; Ma, Z.; Yang, S.; Yao, D.; Zhao, S.; Hu, B.; Tang, G.; Sun, J.; Cheng, M.; Xu, Z.; Wang, Y. Detailed Budget Analysis of HONO in Beijing, China: Implication on Atmosphere Oxidation Capacity in Polluted Megacity. *Atmos. Environ.* **2021**, *244*, 117957. https://doi.org/10.1016/j.atmosenv.2020.117957.

(3) Ren, Y.; Stieger, B.; Spindler, G.; Grosselin, B.; Mellouki, A.; Tuch, T.; Wiedensohler, A.; Herrmann, H. Role of the Dew Water on the Ground Surface in HONO Distribution: A Case Measurement in Melpitz. *Atmos. Chem. Phys. Discuss.* **2020**, *20*, 1–39. https://doi.org/10.5194/acp-2019-1088.

(4) Xue, C.; Zhang, C.; Ye, C.; Liu, P.; Catoire, V.; Le Krysztofiak, G.; Chen, H.; Ren, Y.; Zhao, X.; Wang, J.; Zhang, F.; Zhang, C.; Zhang, J.; An, J.; Wang, T.; Chen, J.; Rg Kleffmann, J.; Mellouki, A.; Mu, Y. HONO Budget and Its Role in Nitrate Formation in the Rural North China Plain. *Cite This Environ. Sci. Technol* **2020**, *54*, 11048–11057. https://doi.org/10.1021/acs.est.0c01832.

(5) Gu, R.; Zheng, P.; Chen, T.; Dong, C.; Wang, Y.; Liu, Y.; Liu, Y.; Luo, Y.; Han, G.; Wang, X.; Zhou, X.; Wang, T.; Wang, W.; Xue, L. Atmospheric Nitrous Acid (HONO) at a Rural Coastal Site in North China: Seasonal Variations and Effects of Biomass Burning. *Atmos. Environ.* **2020**, *229*, 117429. https://doi.org/10.1016/j.atmosenv.2020.117429.

(6) Tang, K.; Qin, M.; Duan, J.; Fang, W.; Meng, F.; Liang, S.; Xie, P.; Liu, J.; Liu, W.; Xue, C.; Mu, Y. A Dual Dynamic Chamber System Based on IBBCEAS for Measuring Fluxes of Nitrous Acid in Agricultural Fields in the North China Plain. *Atmos. Environ.* **2019**, *196*, 10–19. https://doi.org/10.1016/j.atmosenv.2018.09.059.

(7) Meng, F.; Qin, M.; Tang, K.; Duan, J.; Fang, W.; Liang, S.; Ye, K.; Xie, P.; Sun, Y.; Xie, C.; Ye, C.; Fu, P.; Liu, J.; Liu, W. High Resolution Vertical Distribution and Sources of HONO and NO_2 in the Nocturnal Boundary Layer in Urban Beijing, China. *Atmos. Chem. Phys. Discuss.* **2019**, *20*, 1–34. https://doi.org/10.5194/acp-2019-613.

(8) Gil, J.; Kim, J.; Lee, M.; Lee, G.; Lee, D.; Jung, J.; An, J.; Hong, J.; Cho, S.; Lee, J.; Long, R. The Role of HONO in O_3 Formation and Insight into Its Formation Mechanism during the KORUS-AQ Campaign. *Atmos. Chem. Phys. Discuss.* **2019**, 1–30. https://doi.org/10.5194/acp-2019-1012.

(9) Nakashima, Y.; Sadanaga, Y.; Saito, S.; Hoshi, J.; Ueno, H. Contributions of Vehicular Emissions and Secondary Formation to Nitrous Acid Concentrations in Ambient Urban Air in Tokyo in the Winter. *Sci. Total Environ.* **2017**, *592*, 178–186. https://doi.org/https://doi.org/10.1016/j.scitotenv.2017.03.122.

(10) Li, D.; Xue, L.; Wen, L.; Wang, X.; Chen, T.; Mellouki, A.; Chen, J.; Wang, W. Characteristics and Sources of Nitrous Acid in an Urban Atmosphere of Northern China: Results from 1-Yr Continuous Observations. *Atmos. Environ.* **2018**, *182*, 296–306. https://doi.org/10.1016/j.atmosenv.2018.03.033.

(11) Wang, J.; Zhang, X.; Guo, J.; Wang, Z.; Zhang, M. Observation of Nitrous Acid (HONO) in Beijing, China: Seasonal Variation, Nocturnal Formation and Daytime Budget. *Sci. Total Environ.* **2017**, *587–588*, 350–359. https://doi.org/10.1016/j.scitotenv.2017.02.159.

(12) Kasibhatla, P.; Sherwen, T.; Evans, M. J.; Carpenter, L. J.; Reed, C.; Alexander, B.; Chen, Q.; Sulprizio, M. P.; Lee, J. D.; Read, K. A.; Bloss, W.; Crilley, L. R.; Keene, W. C.; Pszenny, A. A. P.; Hodzic, A. Global Impact of Nitrate Photolysis in Sea-Salt Aerosol on NO_x, OH, and O_3 in the Marine Boundary Layer. *Atmos. Chem. Phys.* **2018**, *18*, 11185–11203. https://doi.org/10.5194/acp-18-11185-2018.

(13) Huang, R. J.; Yang, L.; Cao, J.; Wang, Q.; Tie, X.; Ho, K. F.; Shen, Z.; Zhang, R.; Li, G.; Zhu, C.; Zhang, N.; Dai, W.; Zhou, J.; Liu, S.; Chen, Y.; Chen, J.; O'Dowd, C. D. Concentration and Sources of Atmospheric Nitrous Acid (HONO) at an Urban Site in Western China. *Sci. Total Environ.* **2017**, *593–594*, 165–172. https://doi.org/10.1016/j.scitotenv.2017.02.166.

(14) Lee, J. D.; Whalley, L. K.; Heard, D. E.; Stone, D.; Dunmore, R. E.; Hamilton, J. F.; Young, D. E.; Allan, J. D.; Laufs, S.; Kleffmann, J. Detailed Budget Analysis of HONO in Central London Reveals a Missing Daytime Source. *Atmos. Chem. Phys.* **2016**, *16*, 2747–2764. https://doi.org/10.5194/acp-16-2747-2016.

(15) Vandenboer, T. C.; Brown, S. S.; Murphy, J. G.; Keene, W. C.; Young, C. J.; Pszenny, A. A. P.; Kim, S.; Warneke, C.; De Gouw, J. A.; Maben, J. R.; Wagner, N. L.; Riedel, T. P.; Thornton, J. A.; Wolfe, D. E.; Dubé, W. P.; Öztürk, F.; Brock, C. A.; Grossberg, N.; Lefer, B.; Lerner, B.; Middlebrook, A. M.; Roberts, J. M. Understanding the Role of the Ground Surface in HONO Vertical Structure: High Resolution Vertical Profiles during NACHTT-11. *J. Geophys. Res. Atmos.* **2013**, *118*, 10,155-10,171. https://doi.org/10.1002/jgrd.50721.

(16) Oswald, R.; Ermel, M.; Hens, K.; Novelli, A.; Ouwersloot, H. G.; Paasonen, P.; Petäjä,

T.; Sipilä, M.; Keronen, P.; Bäck, J.; Königstedt, R.; Hosaynali Beygi, Z.; Fischer, H.; Bohn, B.; Kubistin, D.; Harder, H.; Martinez, M.; Williams, J.; Hoffmann, T.; Trebs, I.; Sörgel, M. *Atmos. Chem. Phys.* **2015**, *15*, 799–813. https://doi.org/10.5194/acp-15-799-2015.

(17) Michoud, V.; Colomb, A.; Borbon, A.; Miet, K.; Beekmann, M.; Camredon, M.; Aumont, B.; Perrier, S.; Zapf, P.; Siour, G.; Ait-Helal, W.; Afif, C.; Kukui, A.; Furger, M.; Dupont, J. C.; Haeffelin, M.; Doussin, J. F. Study of the Unknown HONO Daytime Source at a European Suburban Site during the MEGAPOLI Summer and Winter Field Campaigns. *Atmos. Chem. Phys.* **2014**, *14*, 2805–2822. https://doi.org/10.5194/acp-14-2805-2014.

(18) Villena, G.; Wiesen, P.; Cantrell, C. A.; Flocke, F.; Fried, A.; Hall, S. R.; Hornbrook, R. S.; Knapp, D.; Kosciuch, E.; Mauldin III, R. L.; McGrath, J. A.; Montzka, D.; Richter, D.; Ullmann, K.; Walega, J.; Weibring, P.; Weinheimer, A.; Staebler, R. M.; Liao, J.; Huey, L. G.; Kleffmann, J. Nitrous Acid (HONO) during Polar Spring in Barrow, Alaska: A Net Source of OH Radicals? *J. Geophys. Res. Atmos.* **2011**, *116* . https://doi.org/https://doi.org/10.1029/2011JD016643.

(19) Spataro, F.; Ianniello, A.; Esposito, G.; Allegrini, I.; Zhu, T.; Hu, M. Occurrence of Atmospheric Nitrous Acid in the Urban Area of Beijing (China). *Sci. Total Environ.* **2013**, *447*, 210–224. https://doi.org/https://doi.org/10.1016/j.scitotenv.2012.12.065.

(20) Song, C. H.; Park, M. E.; Lee, E. J.; Lee, J. H.; Lee, B. K.; Lee, D. S.; Kim, J.; Han, J. S.; Moon, K. J.; Kondo, Y. Possible Particulate Nitrite Formation and Its Atmospheric Implications Inferred from the Observations in Seoul, Korea. *Atmos. Environ.* **2009**, *43*, 2168–2173. https://doi.org/https://doi.org/10.1016/j.atmosenv.2009.01.018.

(21) Su, H.; Cheng, Y. F.; Shao, M.; Gao, D. F.; Yu, Z. Y.; Zeng, L. M.; Slanina, J.; Zhang, Y. H.; Wiedensohler, A. Nitrous Acid (HONO) and Its Daytime Sources at a Rural Site during the 2004 PRIDE-PRD Experiment in China. *J. Geophys. Res.* **2008**, *113*, D14312. https://doi.org/10.1029/2007JD009060.
